# Evaluation of an experimental rat model for comparative studies of bleaching agents

**DOI:** 10.1590/1678-775720150393

**Published:** 2016

**Authors:** Luciano Tavares Angelo Cintra, Francine Benetti, Luciana Lousada Ferreira, Vanessa Rahal, Edilson Ervolino, Rogério de Castilho Jacinto, João Eduardo Gomes, André Luiz Fraga Briso

**Affiliations:** 1 - Univ. Estadual Paulista - UNESP, Faculdade de Odontologia de Araçatuba, Departamento de Endodontia, Araçatuba, SP, Brasil.; 2 - Univ. Estadual Paulista - UNESP, Faculdade de Odontologia de Araçatuba, Departamento de Odontologia Restauradora, Araçatuba, SP, Brasil.; 3 - Univ. Estadual Paulista - UNESP, Faculdade de Odontologia de Araçatuba, Departamento de Ciências Básicas, Araçatuba, SP, Brasil.

**Keywords:** Bleaching agents, Animal models, Hydrogen peroxide

## Abstract

Dental materials, in general, are tested in different animal models prior to their clinical use in humans, except for bleaching agents.

**Objectives:**

To evaluate an experimental rat model for comparative studies of bleaching agents by investigating the influence of different concentrations and application times of H_2_O_2_ gel in the pulp tissue during in-office bleaching of rats’ vital teeth.

**Material and methods:**

The right and left maxillary molars of 50 Wistar rats were bleached with 20% and 35% H_2_O_2_ gels, respectively, for 5, 10, 15, 30, or 45 min (n=10 rats/group). Ten animals (control) were untreated. The rats were killed after 2 or 30 days, and the maxillae were examined by light microscopy. Inflammation was evaluated by histomorphometric analysis with inflammatory cell counting in the coronal and radicular thirds of the pulp. The counting of fibroblasts was also performed. Scores were attributed to the odontoblastic layer and to vascular changes. The tertiary dentin area and the pulp chamber central area were histomorphometrically measured. Data were compared by the analysis of variance and the Kruskal-Wallis test (p<0.05).

**Results:**

After 2 days, the amount of inflammatory cells increased in the occlusal third of the coronal pulp until the time of 15 min for both concentrations of bleaching gels. In 30 and 45 min groups of each concentration, the number of inflammatory cells decreased along with the appearance of necrotic areas. After 30 days, a reduction in the pulp chamber central area and an enlargement of tertiary dentin area were observed without the detection of inflammation areas.

**Conclusion:**

The rat model of extra coronal bleaching showed to be adequate for bleaching protocols studies, as it was possible to observe alterations in the pulp tissues and in the tooth structure caused by different concentrations and periods of application of bleaching agents.

## INTRODUCTION

In-office bleaching with H_2_O_2_ gel is considered a conservative and affordable aesthetic treatment^18^. Its effectiveness is due to the low molecular mass of the main active compound: H_2_O_2_, which easily diffuses through enamel and dentin and releases reactive oxygen species (ROS), thus oxidizing organic structures^2^.

Importantly, H_2_O_2_ and its by-products have varying biological effects on human oral tissues^30^. The ROS-induced oxidative stress can cause mutation, enzyme inactivation, protein degradation, and fragmentation of pulp cells that might manifest as pulpitis and tooth sensitivity^3^. The severity of pulp damage depends on the in-office dental bleaching protocols, and this procedure has been increasingly questioned^2^ ^,^ ^6^ ^,^ ^8^.

An increase in vascular permeability that is dependent of the bleaching procedures duration has been observed in rats’ incisors^12^. A 30 min bleaching session using 35% H_2_O_2_ gel, with or without heat, caused a severe inflammatory reaction in the dental pulp of dogs, including an increased deposition of reparative dentin, the thinning of the odontoblastic layer, inflammatory infiltration, and internal root resorption. Some of these changes, such as inflammation and bleeding, reversed after 60 days^25^. In humans, in-office bleaching of mandibular incisors using the above-mentioned protocol caused partial necrosis in the coronal pulp and a mild inflammatory reaction in the radicular pulp^8^. Moreover, a 45 min bleaching with 35% H_2_O_2_ gel resulted in necrosis near the pulp horns in rats^6^. On the other hand, the application of 38% H_2_O_2_ gel on human premolars did not cause pathological changes in the dental pulp^17^. Therefore, it is evident that the anatomical characteristics of the teeth and of the *in vivo* model analyzed, as well as the bleaching protocols employed, determined different results.

Thus, the lesser thickness of enamel and dentin in teeth of rats might allow a greater penetration of H_2_O_2_, consequently causing more damages to pulp tissues^8^. Therefore, it is essential to characterize the experimental model in rats to find an appropriate protocol to be applied in this model and to allow the performance of further studies on H_2_O_2_ damage to pulp tissues. This model will enable the evaluation of new dosages, formulations, and concentrations of bleaching agents that arise in the market, and also of the potential therapeutic agents that may be used to minimize the damage caused by H_2_O_2_ in different application protocols^6^ ^,^ ^9^.

The choice by the rats in this animal model was due to the easiness in standardizing and controlling these animals, and the possibility of performing other tests^7^ ^,^ ^9^. Thus, it is possible for us to study different variables and to be able to propose, in a second stage and with the results already standardized and evaluated for animals, the validation of the same results in smaller groups of humans by following the ethical principles^9^. Research involving both dogs’ and human’s teeth to study bleaching protocols are impractical because of the difficulty of obtaining the required sample and also because of ethical principles. Furthermore, Cintra, et al*.* ^6^ (2013), when analyzing the influence of the number of bleaching sessions in pulp tissues, indicated the possibility of using teeth of rats for the study of bleaching protocols. Using the rat model for studying bleaching agents is relatively simple and easy to reproduce.

Therefore, the purpose of this study was to characterize an experimental animal model for comparative studies of bleaching agents by investigating the influence of different concentrations and application times of H_2_O_2_ gel during in-office bleaching of rats’ vital teeth. We hypothesized that: (I) the H_2_O_2_ bleaching gel is capable of penetrating the pulp tissue and causing greater damages when the time of application and the concentration of H_2_O_2_ are increased; (II) the pulp tissue is capable of recovering from the damages caused by H_2_O_2_ after long periods of time.

## MATERIAL AND METHODS

### Animals

Sixty male Wistar rats (180-200 g) were used in this study. The animals were housed in a temperature-controlled environment (22°C ± 1°C) in a standard light-dark schedule with unrestricted access to food and water. The experimental protocol was approved by the Ethics Committee (CEUA 2013-01253) and conducted according to Guide for the Care and Use of Laboratory Animals of the National Institutes of Health (Bethesda, Maryland, USA).

### Tooth bleaching

The rats were anesthetized with intramuscular injections of ketamine (87 mg/kg; Francotar, Virbac do Brasil Indústria e Comércio Ltda., Roseira, São Paulo, Brazil) and xylazine (13 mg/kg; Rompum, Bayer SA, São Paulo, São Paulo, Brazil). The right and left molars in every animal were bleached with 20% (Whiteness HP Blue, FGM Dental Products, Joinville, Santa Catarina, Brazil) and 35% H_2_O_2_ (Whiteness HP Maxx, FGM Dental Products, Joinville, Santa Catarina, Brazil), respectively, for 5, 10, 15, 30, or 45 min (n=10 rats/group). Ten animals (controls) did not receive any treatment.

### Histology

Animals were killed by the anesthetic solution overdose 2 or 30 days after the bleaching sessions. Their bilateral maxillae were separated, dissected, and fixed in a 10% buffered formalin solution for 24 h. Specimens were decalcified in a 10% EDTA solution for three months and then dehydrated in a graded ethanol series, embedded in paraffin, cut into 6 μm sagittal cross-sections, and stained with hematoxylin and eosin (H&E). The serial histological sections of each specimen were selected from the point where the mesial root of the first molar was seen in all its longitudinal extension.

The coronal pulp was divided into occlusal, middle, and cervical thirds and the radicular pulp was divided into cervical, middle, and apical thirds^6^. Inflammation was evaluated by histomorphometric analysis with inflammatory cells counting in the coronal and radicular thirds of the pulp. The counting of fibroblasts was also performed. The cell counting was performed in a 10 μm^2^ field in each third of the pulp tissue of each specimen, examined under light microscopy (×1000 magnification; DM4000 B, Leica Microsystems, Wetzlar, Germany).

Scores were attributed to the odontoblastic layer in each third of the pulp tissue as follows: 1- intact odontoblastic layer; 2- disorganized odontoblastic layer; or 3- disruption of the odontoblastic layer. Scores to vascular changes were also assigned as follows: 1- normality; 2- increase in the number of blood vessels; or 3- necrosis.

**Figure 1:**
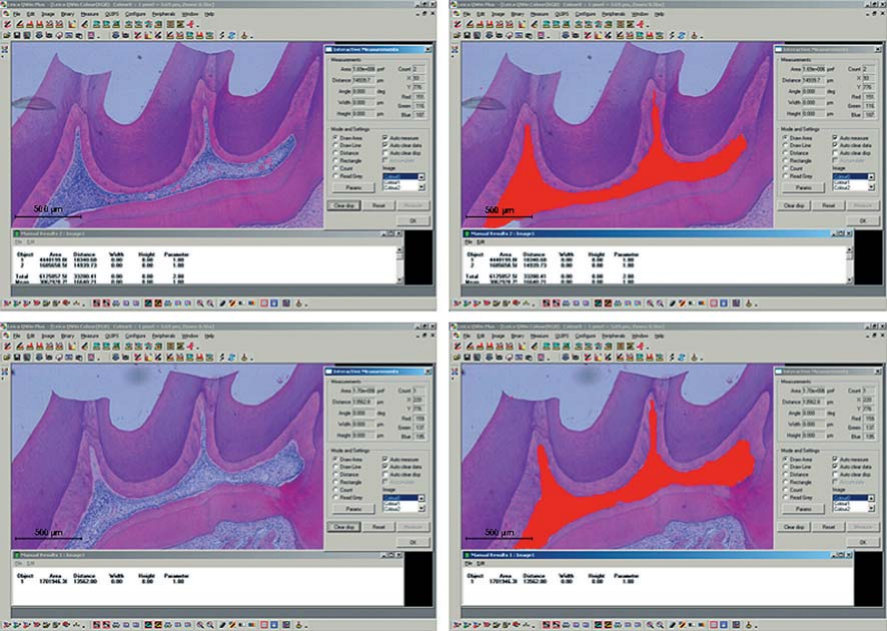
Measurement of the central area of the pulp chamber in the experimental groups using the Leica QWin V3 Image Processing and Analysis Software. The values obtained were analyzed by the Kolmogorov-Smirnov normality test and the One-way ANOVA test (p<0.05)

The mean of central area of the pulp chamber was measured by image processing software (Leica QWin V3, Leica Microsystems, Wetzlar, Germany) (Figure 1). By taking into account the obtained values it was possible to calculate the reduction percentage in the central area of the pulp chamber in the treated groups considering the central area of the control group.

After the application of the Kolmogorov-Smirnov test of normality, the data obtained in the counting of inflammatory cells and fibroblasts were submitted to the Two-way analysis of variance and Tukey tests for intergroup comparisons at a significance level of 5% (p<0.05). The scores obtained in the analysis of the odontoblastic layer and of vascular changes were submitted to the Kruskal-Wallis and Dunn tests (p<0.05). The values obtained in the mean of central area of the pulp chamber were submitted to Kolmogorov-Smirnov test of normality and One-way analysis of variance test (p<0.05).

## RESULTS

### Inflammatory response

#### Control group

**Figure 2:**
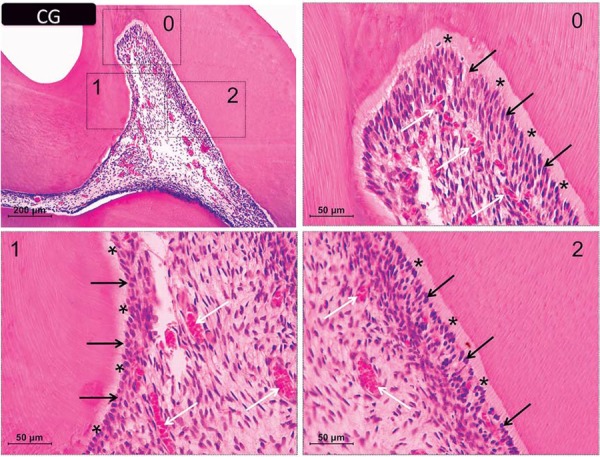
Representative images of H&E-stained sections with controls’ coronal pulp. Panels 0, 1, and 2 are magnified images (×400) of the respective insets in the upper left panel (×100 magnification). Black arrows indicate the odontoblastic layer and white arrows show the distribution of cells and blood vessels in the subjacent tissue. Asterisks show the predentin layer. H&E=hematoxylin & eosin

The dental pulp of control animals exhibited well-defined acellular and cell-rich layers under an intact odontoblastic layer and even distribution of cells, blood vessels, and extracellular matrix structures (Figure 2).

#### 20%-5 min group

**Figure 3:**
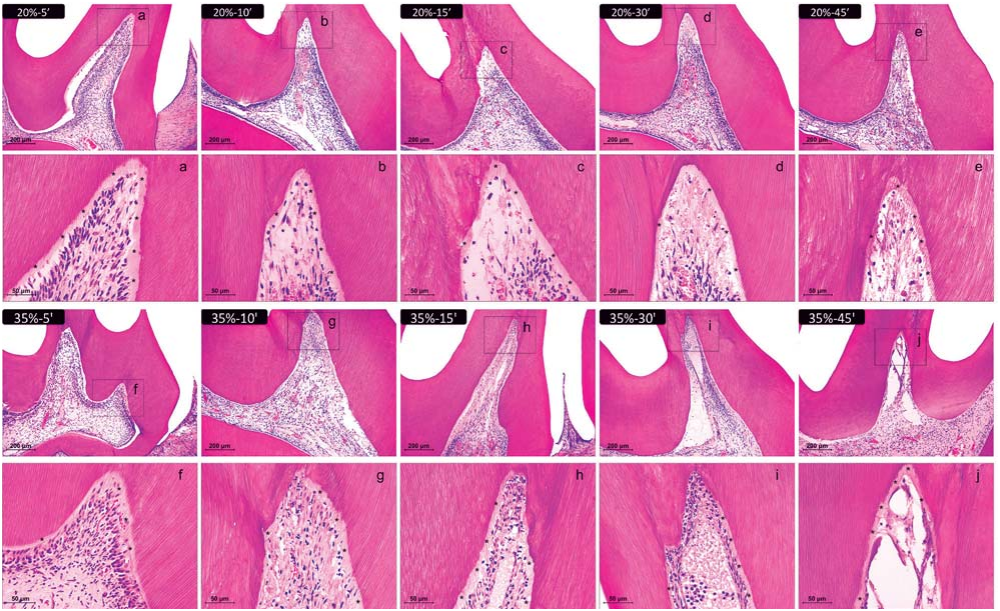
Representative images of H&E-stained sections showing the coronal pulp 2 days after bleaching. Panels a, b, c, d, and e represent the groups treated with 20% H2O2 gel and panels f, g, h, i, and j represent those treated with 35% H2O2 gel for 5, 10, 15, 30, and 45 min, respectively (×100 magnification). Panels a-j are magnified images (×400) of the insets in panels a-j, respectively. The asterisks show the predentin layer. The number of inflammatory cells and fibroblasts was obtained in each third of the pulp tissue (at 1000x magnification) and subjected to the Kolmogorov-Smirnov normality test, the Two-way ANOVA and Tukey test (p<.05); and the scores to odontoblastic layer and vascular changes to Kruskal-Wallis and Dunn test (p<0.05). H&E=hematoxylin & eosin

This group exhibited no inflammatory infiltrate. The dental pulp appeared to be similar to that in the control group. The odontoblastic layer was intact and blood vessels showed normal characteristics. The cementum, periodontal ligament, alveolar bone, and other supporting structures also appeared to be normal (Figure 3A).

#### 20%-10 min group

This group did not exhibit a considerable amount of inflammatory cells. There was a reduction in the amount of fibroblasts in the occlusal and middle thirds of the coronal pulp. The odontoblastic layer was partially disorganized in the occlusal third, and there was an increase in the number of blood vessels in the occlusal and middle thirds of the coronal pulp (Figure 3B).

#### 20%-15 min group

There was an increase in the amount of inflammatory cells in the occlusal and middle thirds of the coronal pulp, a reduction in the amount of fibroblasts, and an increase in the amount of blood vessels. The odontoblastic layer was partially disorganized in the occlusal third (Figure 3C).

#### 20%-30 min group

In this group, the higher number of inflammatory cells was found in the middle third of the coronal pulp. There was a large reduction in the amount of fibroblasts in the occlusal third, where there was disruption of the odontoblastic layer. The amount of blood vessels increased in the occlusal and middle thirds of the coronal pulp (Figure 3D). The radicular pulp appeared to be normal in all cases.

#### 20%-45 min group

This group showed increased number of inflammatory cells in the cervical and middle thirds of the coronal pulp. The occlusal third showed necrotic areas. A reduction in the number of fibroblasts was observed in the cervical third of the crown. The odontoblastic layer was absent in the occlusal third and partly disorganized in the middle third of the coronal pulp. There was an insignificant amount of inflammatory cells in the cervical third of the radicular pulp (Figure 3E).

#### 35%-5 min group

The amount of inflammatory cells in this group was not significant. The amount of fibroblasts reduced in the occlusal third of the coronal pulp, where the odontoblastic layer was partially disorganized. An increase in the number of blood vessels was observed in all areas of the coronal pulp (Figure 3F).

#### 35%-10 min group

In this group, there was an increase in the number of inflammatory cells in the occlusal and middle thirds of the coronal pulp. The amount of fibroblasts reduced in the occlusal third. The odontoblastic layer was absent in the occlusal third and was partly disorganized in the middle third of the coronal pulp. There was an increase in the number of blood vessels throughout the coronal pulp (Figure 3G).

#### 35%-15 min group

There was an increase in the number of inflammatory cells in the occlusal and middle thirds of the coronal pulp, while the number of fibroblasts reduced. The odontoblastic layer was absent in the occlusal third, and partly disorganized in the middle third of the coronary pulp. There was an increase in the number of blood vessels throughout the coronal pulp (Figure 3H).

#### 35%-30 min group

The number of inflammatory cells and fibroblasts reduced in the occlusal third, where there was the presence of necrotic areas. There was an increase in the number of inflammatory cells in the middle and cervical thirds of the coronary pulp. The amount of fibroblasts was still low in the middle third of the coronary pulp. The odontoblastic layer was absent in occlusal and middle thirds of the crown. There was an increase in the number of blood vessels in the middle and cervical thirds of the coronal pulp. The occlusal third were characterized as necrosis. A small amount of inflammatory cells was found in the cervical third of the radicular pulp (Figure 3I).

#### 35%-45 min group

There was necrosis in the occlusal third of this group with absence of inflammatory cells and fibroblasts. The number of inflammatory cells increased in the cervical and middle thirds of the coronal pulp, and in the cervical third of the radicular pulp. The number of fibroblasts reduced in these thirds. There was no odontoblastic layer in the occlusal and middle thirds of the crown, and this layer was partially disorganized in the cervical third. The number of blood vessels increased in the cervical third of coronary and radicular pulps. The remaining thirds seemed to be normal (Figure 3J).

## Reparative Dentin Area

**Figure 4:**
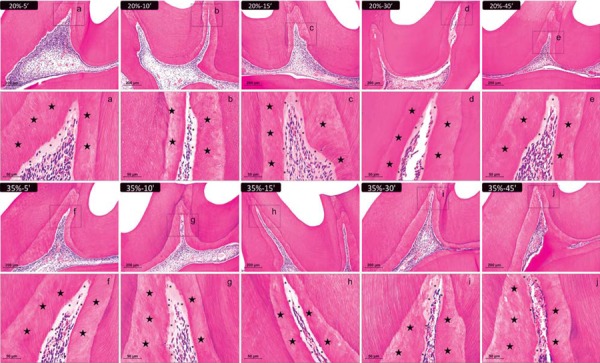
Representative images of H&E-stained sections showing the coronal pulp 30 days after bleaching. Panels a, b, c, d, and e represent the groups treated with 20% H2O2 gel and panels f, g, h, i, and j represent those treated with 35% H2O2 gel for 5, 10, 15, 30, and 45 min, respectively (×100 magnification). Panels a-j are magnified images (×400) of the insets in panels a-j, respectively. The stars indicate the reparative dentin layer; the asterisks show the predentin layer. The values of the area of the pulp chamber were obtained, as shown in Figure 1, to carry out the statistical analysis. H&E=hematoxylin & eosin

Thirty days after the bleaching sessions, all specimens showed normal dental pulp. However, the central area of the pulp chamber reduced, and the tertiary dentin area increased (Figure 4).

## Intergroup Comparisons

Table 1 shows the amount of inflammatory cells of all experimental groups. The most predominant inflammatory cells found were mononuclear cells, such as lymphocytes, macrophages, and plasmocytes, characterizing a chronic inflammatory infiltrate. The amount of inflammatory cells grew gradually with the increasing concentrations and the application time of the bleaching gel, up to the 15 min application groups of each bleaching gel, in the occlusal third of the coronal pulp. The groups that received the application of 30 and 45 min of each bleaching agent showed areas of necrosis in the occlusal third with a decrease in the amount of inflammatory cells. Significant differences were observed between the bleached groups and the control group in the occlusal third (p<0.05), except for the 35%-45 min group, with absence of cells. Significant differences in the middle third of the coronal pulp were noted between the control group and the 20%-10 to 45 min and the 35%-5 to 45 min groups (p<0.05). In the cervical third, the difference with the control group remained in 20%-15 to 45 min and 35%-10 to 45 min groups (p<0.05). In the cervical third of the radicular pulp, significant differences were noted between the 20%-45 min and the 35%-30 min groups with the other groups and 35%-45 min with all groups. Significant differences were not observed in the other radicular thirds (p>0.05).

**Table 1:** Inflammatory cell count (*per* 10 μm2) in the pulp thirds of each group (Mean ±SD)

Group		Coronal			Radicular		
		Occlusal	Middle	Cervical	Cervical	Middle	Apical
Control		0.0 ±0.0^a^	0.0 ±0.0^a^	0.0 ±0.0^a^	0.0 ±0.0^a^	0.0 ±0.0^a^	0.0 ±0.0^a^
20% H_2_O_2_ gel	5 min	3.6 ±0.5^b^	2.2 ±0.4^ab^	1.0 ±0.4^ab^	0.0 ±0.0^a^	0.0 ±0.0^a^	0.0 ±0.0^a^
	10 min	5.6 ±1.1^bc^	4.6 ±1.1^bcd^	2.2 ±0.5^abc^	0.0 ±0.0^a^	0.0 ±0.0^a^	0.0 ±0.0^a^
	15 min	7.6 ±1.5^c^	6.0 ±1.4^ce^	3.4 ±0.9^bd^	0.0 ±0.0^a^	0.0 ±0.0^a^	0.0 ±0.0^a^
	30 min	5.2 ±0.8^bc^	8.2±1.3^e^	5.2 ±1.1^d^	0.0 ±0.0^a^	0.0 ±0.0^a^	0.0 ±0.0^a^
	45 min	3.2 ±0.8^b^	13.0 ±2.9^fg^	9.0 ±2.3^e^	5.6 ±0.9^b^	0.0 ±0.0^a^	0.0 ±0.0^a^
35% H_2_O_2_ gel	5 min	4.0 ±0.7^b^	4 ±1.0^bc^	2.2 ±0.5^abc^	0.0 ±0.0^a^	0.0 ±0.0^a^	0.0 ±0.0^a^
	10 min	10.6±2.3^d^	7.4 ±1.1^de^	4.4 ±1.1^cd^	0.0 ±0.0^a^	0.0 ±0.0^a^	0.0 ±0.0^a^
	15 min	14.6±3.6^e^	11.8±2.3^f^	5.2 ±1.3^d^	0.0 ±0.0^a^	0.0 ±0.0^a^	0.0 ±0.0^a^
	30 min	6.0±1.4^bc^	15.6±1.3^g^	9.8 ±2.6^e^	5.8 ±0.8^b^	0.0 ±0.0^a^	0.0 ±0.0^a^
	45 min	0.0 ±0.0^a^	12.0±2.9^f^	11.0±2.6^e^	7.6±1.5^c^	0.0 ±0.0^a^	0.0 ±0.0^a^

*Different letters in the columns indicate significant difference between the groups (Kolmogorov-Smirnov normality test and the Two-way ANOVA and Tukey test - p<0.05).

Table 2 shows the amount of fibroblasts of the experimental groups. The 20%-10 to 45 min, and 35% groups showed a significant decrease in the number of fibroblasts in the occlusal third compared to the control group (p<0.05). This decrease remained in the middle third of the coronal pulp in the 20%-15 to 45 min, and 35%-15 to 45 min groups (p<0.05). In the cervical third, only the groups that received the bleaching gels for 45 min showed a significant difference of the control group (p<0.05). Significant differences were not observed in the radicular thirds of any group (p>0.05).

**Table 2:** Fibroblasts count (*per* 10 μm2) in the pulp thirds of each group (Mean ±SD)

Group		Coronal			Radicular		
		Occlusal	Middle	Cervical	Cervical	Middle	Apical
Control		65.2 ±8.7^a^	67.0 ±7.8^ab^	44.0 ±4.6^abc^	38.0 ±3.7^a^	33.8 ±2.9^a^	37.2 ±3.3^a^
20% H_2_O_2_ gel	5 min	51.8 ±6.6^ab^	72.2 ±11.9^a^	52.8 ±8.5^bc^	39.4 ±4.2^a^	35.8 ±3.0^a^	37.0 ±2.3^a^
	10 min	45.2 ±9.2^bc^	54.0 ±4.2^bc^	55.6 ±11.2^c^	36.8 ±2.9^a^	37.6 ±3.1^a^	35.0 ±3.2^a^
	15 min	36.2 ±3.6^c^	46.4 ±2.4^cd^	43.2 ±8.4^ac^	41.2 ±0.8^a^	38.2 ±3.6^a^	35.2 ±3.3^a^
	30 min	5.8 ±1.6^de^	32.4±7.5^de^	34.6 ±5.5^ad^	39.6 ±1.9^a^	36.2 ±3.8^a^	37.2 ±2.2^a^
	45 min	0.0 ±0.0^d^	26.0 ±1.6^efg^	29.0 ±8.2^def^	36.0 ±4.5^a^	37.6 ±4.2^a^	38.2 ±2.6^a^
35% H_2_O_2_ gel	5 min	15.2 ±2.8^e^	64.8 ±11.3^ab^	40.8 ±8.0^abe^	35.2 ±2.9^a^	34.8 ±3.9^a^	39.0 ±2.3^a^
	10 min	13.2±3.0^de^	54.6±12.4^abc^	35.2 ±3.6^ae^	38.6 ±1.5^a^	36.2 ±4.3^a^	37.6 ±3.6^a^
	15 min	10.8±2.9^de^	41.6±9.7^cdf^	34.2 ±3.0^ae^	37.6 ±2.5^a^	38.8 ±1.6^a^	37.6 ±4.3^a^
	30 min	5.8±0.8^de^	23.2±5.0^eg^	30.4 ±4.0^aef^	36.0 ±4.2^a^	37.8 ±2.2^a^	39.6 ±4.5^a^
	45 min	0.0 ±0.0^d^	10.2±2.7^g^	19.6±1.8^f^	38.0±2.7^a^	36.8 ±2.6^a^	40.2 ±4.2^a^

*Different letters in the columns indicate significant difference between the groups (Kolmogorov-Smirnov normality test and the Two-way ANOVA and Tukey test - p<0.05).

Table 3 shows the scores assigned to the odontoblast layer of each experimental group. Significant differences were observed between the 20%-45 min, 35%-30 and 45 min in the occlusal third compared to the control and the 20%-5 min groups (p<0.05). In the middle third of the coronal pulp, the difference remained between the 35%-45 min group with the control and 20%-5 to 30 min groups (p<0.05). There were no significant differences in the cervical third and in radicular thirds (p>0.05).

**Table 3:** Comparison of the Odontoblastic Layer Scores (Median)

Group		Coronal			Radicular		
		Occlusal	Middle	Cervical	Cervical	Middle	Apical
Control		1^a^	1^a^	1^a^	1^a^	1^a^	1^a^
20% H_2_O_2_ gel	5 min	1^a^	1^a^	1^a^	1^a^	1^a^	1^a^
	10 min	2^ab^	1^a^	1^a^	1^a^	1^a^	1^a^
	15 min	2^ab^	1^a^	1^a^	1^a^	1^a^	1^a^
	30 min	3^ab^	1^a^	1^a^	1^a^	1^a^	1^a^
	45 min	3^b^	2^ab^	1^a^	1^a^	1^a^	1^a^
35% H_2_O_2_ gel	5 min	2^ab^	1^ab^	1^a^	1^a^	1^a^	1^a^
	10 min	3^ab^	2^ab^	1^a^	1^a^	1^a^	1^a^
	15 min	3^ab^	2^ab^	1^a^	1^a^	1^a^	1^a^
	30 min	3^b^	3^ab^	1^a^	1^a^	1^a^	1^a^
	45 min	3^b^	3^b^	2^a^	1^a^	1^a^	1^a^

*Different letters in the columns indicate significant difference between the groups (Kruskal-Wallis and Dunn tests - p<0.05).

Table 4 shows the scores assigned to the vascular changes of each experimental group. Significant differences were observed between the 20%-45 min and 35%-45 min in the occlusal third compared to the control and 20%-5 min groups (p*<*0.05). In the middle third of the coronal pulp, the difference remained between the 35%-45 min group with the control and 20%-5 groups (p*<*0.05). There was no significant difference in the cervical third and in radicular thirds (p>0.05).

**Table 4:** Comparison of the Vascular Changes Scores (Median)

Group		Coronal			Radicular		
		Occlusal	Middle	Cervical	Cervical	Middle	Apical
Control		1^a^	1^a^	1^a^	1^a^	1^a^	1^a^
20% H_2_O_2_ gel	5 min	1^a^	1^a^	1^a^	1^a^	1^a^	1^a^
	10 min	2^ab^	2^ab^	1^a^	1^a^	1^a^	1^a^
	15 min	2^ab^	2^ab^	1^a^	1^a^	1^a^	1^a^
	30 min	2^ab^	2^ab^	1^a^	1^a^	1^a^	1^a^
	45 min	3^b^	2^ab^	1^a^	1^a^	1^a^	1^a^
35% H_2_O_2_ gel	5 min	2^ab^	2^ab^	2^a^	1^a^	1^a^	1^a^
	10 min	2^ab^	2^ab^	2^a^	1^a^	1^a^	1^a^
	15 min	2^ab^	2^ab^	2^a^	1^a^	1^a^	1^a^
	30 min	3^ab^	2^ab^	2^a^	1^a^	1^a^	1^a^
	45 min	3^b^	3^b^	2^a^	1^a^	1^a^	1^a^

*Different letters in the columns indicate significant difference between the groups (Kruskal-Wallis and Dunn tests - p<0.05).

After 30 days, the specimens showed a gradual increase in the tertiary dentin area. Significant differences were observed between the 35%-45 min and the other groups, except for the 35%-30 min group (p<0.05). The groups 20%-5 min, 20%-10 min, 20%-15 min, and 35%-5 min did not differ significantly from the control group (p>0.05) (Table 5).

**Table 5:** Change in Pulp Chamber Central Area (μm2)

Group		Mean (105)*	SD (105)	% reduction
Control		18.46^a^	2.38	0.00
20% H_2_O_2_ gel	5 min	17.57^a^	2.30	4.82
	10 min	17.18^a^	1.85	6.93
	15 min	15.65^ab^	1.78	15.22
	30 min	13.47^bc^	1.43	27.03
	45 min	11.23^cd^	1.17	39.16
35% H^2^O_2_ gel	5 min	14.61^ab^	0.46	20.85
	10 min	13.41^bc^	0.72	27.36
	15 min	12.56^bcd^	0.71	32.33
	30 min	10.01^de^	0.62	45.77
	45 min	6.98^e^	0.51	62.18

*Different letters in the column indicate significant difference (Kolmogorov-Smirnov normality test and the One-way ANOVA test - p<0.05).

## DISCUSSION

Tooth bleaching is an aesthetic alternative for discolored teeth, but it has potential adverse effects that are not completely understood yet^28^. A single bleaching session can produce significant esthetics results, but longer application times and multiple sessions may be required for optimal outcomes, increasing the risk of tooth sensitivity^20^ and pulp damage^6^.

A large number of *in vitro* studies have shown the ROS generated by H_2_O_2_ of bleaching gels is capable of causing histochemical and morphological changes in enamel and dentin^4^ ^,^ ^5^.

*In vivo* studies showed cellular damage, classified as mild to severe. These include studies performed in dogs^25^ ^,^ ^26^, human mandibular incisors^8^ ^,^ ^19^, and in rat incisors^12^ ^,^ ^13^ and molars^6^ ^.^ Studies on cell cultures also demonstrated cellular damage not only to DNA cells^23^ but also such as apoptosis^14^, inflammation^3^, cytotoxicity^30^, cell viability reduction^27^, or the ageing of the dental pulp^1^ ^,^ ^28^. The cytotoxicity of the bleaching gel was also observed in this study.

Studies predominantly with *ex vivo* manipulated cells have significant importance in the preliminary studies on bleaching agents. However, in those studies, pulp cells are not examined as organized tissues. Teeth have vital pulp components that can prevent or hinder the H_2_O_2_ effects in pulp tissues, as dentinal fluid, cytoplasmatic extensions^17^ ^,^ ^30^, and antioxidant enzymes as superoxide dismutase and catalase that promote the enzymatic degradation of H_2_O_2_ ^11^ ^,^ ^17^. Therefore, *in vivo* experiments are the ones that best represent the reality of bleaching effects.

The application of 38% H_2_O_2_ gel in human premolars does not cause pathological changes in the dental pulp^17^. However, the application of the same concentration in human mandibular incisors causes necrosis in the coronal pulp in a similar way to that observed in rats’ molars^6^, possibly because of the thinner enamel and dentin^8^. These findings indicate the morphological characteristics of different tooth structures influence directly the pulp damage. Ideally, upper anterior human teeth should be used to determine the pulp changes accurately. Even under these conditions, other factors would influence the results such as age, presence of restorations, previous trauma, among others.

Even though variations of the pulp response have been shown in human teeth, our study aimed to characterize an experimental animal model of ease reproduction and standardization to the study of new bleaching agents, posology, concentrations, and application time.

In dogs’ teeth, dental bleaching using 35% H_2_O_2_ showed greater changes immediately beneath the region where the gel was applied^27^, similarly to that observed in groups of 35% H_2_O_2_ gel applied for 30 or 45 min, and 20% H_2_O_2_ gel for 45 min in this study. Severe pulp damage may occur when bleaching agents are applied in the buccal surface of teeth with thin enamel and dentin^8^ ^,^ ^25^ ^,^ ^26^. Dog’s teeth present difficulties in standardization and there is an insufficient number of similar teeth for new studies. Furthermore, nowadays, studies in dogs have been avoided by ethical reasons.

The use of rats in the experimental model presents advantages such as straightforwardness in the handling and in the reproduction, and mainly, the easiness for controlling, predicting^22^, and standardizing^6^. Moreover, this model is better accepted regarding ethical and economic concerns^9^.

Despite the difference in enamel and dentin thickness between humans’ and rats’ teeth (2.5 mm *vs*. 100 µm, respectively), they both show the same proportion of these structures^6^ ^,^ ^9^. In addition, rats’ molars have anatomical, histological, biological, and physiological features similar to human molars^9^ ^,^ ^24^. Also, rat molars exhibit the same structural characteristics of the pulp chamber and of the pulp tissues, where the essential biological reactions and the wound healing of rats’ molar teeth are comparable to that of other mammals^9^. Conversely, rats’ incisors are typical of rodents, having permanent growth, with a wide-open apex, and cannot be compared to human teeth^9^.

In the present study, 35% H_2_O_2_ gel applied for 30 or 45 min caused necrosis or a severe inflammatory response in the dental pulp, especially in the upper coronal two-thirds. Clinically, in-office bleaching with high H_2_O_2_ concentrations for 30-45 min in a single session is frequently associated with a high incidence of tooth sensitivity^8^. Considering the similarity of the results found in this study with the results of Costa, et al*.* ^8^ (2010), we suggested that rat molars can be targeted and improved as an experimental model to predict the results of procedures performed in human mandibular incisors with the same concentration and duration of application^6^.

The amount of H_2_O_2_ detected in the pulp chamber is related to the concentration and application time of the gel^2^. The use of 35% H_2_O_2_ gel applied for 30 min, and 20% and 35% H_2_O_2_ gel for 45 min was related to changes in the vascular permeability^12^. Therefore, our study was carried out with several application times and two concentrations, one of which more commonly employed at the clinic (H_2_O_2_ 35%)^16^ ^,^ ^29^, and another with lower concentration (H_2_O_2_ 20%). Our results allow us to choose the concentration and the time of application to analyze comparatively the initial inflammatory process (after two days) as well as in the subsequent reparative process (after 30 days).

In our evaluation by 30 days after bleaching, we observed that all groups showed signs of repair. Tertiary dentin was formed to protect the dental pulp, reducing pulp chamber central area. Inflammatory cells were absent. The low-concentration/application time groups showed significant differences as to compare the high-concentrations/application time groups. Studies of the effects of high concentrations of bleaching gels on pulp cell cultures have shown that products released by 35% H_2_O_2_ gel can diffuse through enamel and dentin and cause significant cell damage^10^ ^,^ ^30^.

Cell membrane can be penetrated by H_2_O_2_, increase alkaline phosphatase activity, induce apoptosis in the periodontal ligament and dental pulp^15^ as well as stimulate mineralization^21^. Increased alkaline phosphatase activity and extracellular matrix mineralization reveal dentin production^28^. The model in rats can also be used in long term analysis to determine clinical protocols of application that produce less pulp damages along time.

The characterization of this experimental model does not replace human trials, but allows us to know the action mechanisms of new bleaching agents; to compare bleaching protocols; and to study desensitizing and remineralizing agents used before and after bleaching to minimize its effects on pulp tissues.

Therefore, the rat model of extra coronal bleaching showed to be adequate to study bleaching protocols, as it was possible to observe alterations in the pulp tissues and in tooth structure caused by different concentrations and periods of application of bleaching agents. In-office bleaching with H_2_O_2_ gel causes immediate inflammation and accelerates the aging of the dental pulp by inducing the deposition of the tertiary dentin, and the degree of damage grew with the increasing concentrations and application times of the bleaching agent.

## References

[1] Arai M, Shibata Y, Pugdee K, Abiko Y, Ogata Y (2007). Effects of reactive oxygen species (ROS) on antioxidant system and osteoblastic differentiation in MC3T3-E1 cells. IUBMB Life.

[2] Benetti AR, Valera MC, Mancini MN, Miranda CB, Balducci I (2004). In vitro penetration of bleaching agents into the pulp chamber. Int Endod J.

[3] Bhattacharyya S, Dudeja PK, Tobacman JK (2008). Carrageenan-induced NFkappaB activation depends on distinct pathways mediated by reactive oxygen species and Hsp27 or by Bcl10. Biochim Biophys Acta.

[4] Borges AB, Torres CR, Souza PA, Caneppele TM, Santos LF, Magalhães AC (2012). Bleaching gels containing calcium and fluoride: effect on enamel erosion susceptibility. Int J Dent.

[5] Chen HP, Chang CH, Liu JK, Chuang SF, Yang JY (2008). Effect of fluoride containing bleaching agents on enamel surface properties. J Dent.

[6] Cintra LTA, Benetti F, Facundo AC, Ferreira LL, Gomes JE, Ervolino E (2013). The number of bleaching sessions influences pulp tissue damage in rat teeth. J Endod.

[7] Costa CA, Oliveira MF, Giro EM, Hebling J (2003). Biocompatibility of resin-based materials used as pulp-capping agents. Int Endod. J.

[8] Costa CAS, Riehl H, Kina JF, Sacono NT, Hebling J (2010). Human pulp responses to in-office tooth bleaching treatment. Oral Surg Oral Med Oral Pathol Oral Radiol Endod.

[9] Dammaschke T (2010). Rat molar teeth as a study model for direct pulp capping research in dentistry. Lab Anim.

[10] Duncan HF, Smith AJ, Fleming GJ, Cooper PR (2012). Histone deacetylase inhibitors induced differentiation and accelerated mineralization of pulp-derived cells. J Endod.

[11] Esposito P, Varvara G, Murmura G, Terlizzi A, Caputi S (2003). Ability of healthy andinflamed human dental pulp to reduce hydrogen peroxide. Eur J Oral Sci.

[12] Ferreira VG, Nabeshima CK, Marques MM, Paris AF, Gioso MA, Reis RS (2013). Tooth bleaching induces changes in the vascular permeability of rat incisor pulps. Am J Dent.

[13] Frigo L, Pallota RC, Meneguzzo D, Marcos RL, Penna SC, Lopes-Martins RA (2009). Avaliação do efeito da clareação dentária fotoativada sobre a polpa dentária em modelo experimental de ratos. Rev Dental Press Estet.

[14] Han YH, Kim SZ, Kim SH, Park WH (2008). Pyrogallol as a glutathione depletor induces apoptosis in HeLa cells. Int J Mol Med.

[15] Hanks CT, Fat JC, Wataha JC, Corcoran JF (1993). Cytotoxicity and dentin permeability of carbamide peroxide and hydrogen-peroxide vital bleaching materials, in vitro. J Dent Res.

[16] Joiner A (2006). The bleaching of teeth: a review of the literature. J Dent.

[17] Kina JF, Huck C, Riehl H, Martinez TC, Sacono NT, Ribeiro AP (2010). Response of human pulps after professionally applied vital tooth bleaching. Int Endod J.

[18] Marson FC, Gonçalves RS, Silva CO, Cintra LT, Pascotto RC, Santos PH (2015). Penetration of hydrogen peroxide and degradation rate of different bleaching products. Oper Dent.

[19] Marson FC, Guedes AA, Camargo WR, Progiante PS, Silva CO (2014). The gel cytotoxicity in relation to the dental pulp. J Surg Clin Dent.

[20] Marson FC, Sensi LG, Vieira LC, Araújo E (2008). Clinical evaluation of in-office dental bleaching treatments with and without the use of light-activation sources. Oper Dent.

[21] Matsui S, Takahashi C, Tsujimoto Y, Matsushima K (2009). Stimulatory effects of low-concentration reactive oxygen species on calcification ability of human dental pulp cells. J Endod.

[22] Penna LA, Rode SM (2000). Estudo morfológico da polpa de molares de ratos Wistar frente a uma oclusão traumática experimental. Pesq Odont Bras.

[23] Sanz A, Gómez J, Caro P, Barja G (2006). Carbohydrate restriction does not change mitochondrial free radical generation and oxidative DNA damage. J Bioenerg Biomembr.

[24] Sasaki T, Kawamata-Kido H (1995). Providing an environment for reparative dentine induction in amputated rat molar pulp by high molecular-weight hyaluronic acid. Arch Oral Biol.

[25] Seale NS, McIntosh JE, Taylor AN (1981). Pulpal reaction to bleaching of teeth in dogs. J Dent Res.

[26] Seale NS, Wilson CF (1985). Pulpal response of bleaching of teeth in dogs. Pediatr Dent.

[27] Soares DG, Pontes EC, Ribeiro AP, Basso FG, Hebling J, Costa CA (2013). Low toxic effects of a whitening strip to cultured pulp cells. Am J Dent.

[28] Soares DGS, Ribeiro APD, Sacono NT, Coldebella CR, Hebling J, Costa CA (2011). Transenamel and transdentinal cytotoxicity of carbamide peroxide bleaching gels on odontoblast-like MDPC-23 cells. Int Endod J.

[29] Sulieman M, Addy M, Macdonald E, Rees JS (2005). The bleaching depth of a 35% hydrogen peroxide based in-office product: a study in vitro. J Dent.

[30] Trindade FZ, Ribeiro AP, Sacono NT, Oliveira CF, Lessa FC, Hebling J (2009). Trans-enamel and trans-dentinal cytotoxic effects of a 35% H2O2 bleaching gel on cultured odontoblast cell lines after consecutive applications. Int Endod J.

