# ERRATUM

**DOI:** 10.1590/1678-7757-2025er003

**Published:** 2025-06-27

Due to a publishing error the article: “**Evaluation of an experimental rat model for comparative studies of bleaching agents**”, published at Journal of Applied Oral Science 2016;24(1):95-104. doi: 10.1590/1678-775720150393 was published with the following error:

Where it reads:

**DOI:**

http://dx.doi.org/10.1590/1678-775720150393

It should read:

**DOI:**

http://dx.doi.org/10.1590/1678-775720150393ar

